# Repurposing ^18^F-FMISO as a PET tracer for translational imaging of nitroreductase-based gene directed enzyme prodrug therapy

**DOI:** 10.7150/thno.55092

**Published:** 2021-04-07

**Authors:** Gorka Ruiz de Garibay, Elvira García de Jalón, Endre Stigen, Kjetil B Lund, Mihaela Popa, Ben Davidson, Mireia Mayoral Safont, Cecilie B. Rygh, Heidi Espedal, Torill M Barrett, Bengt Erik Haug, Emmet McCormack

**Affiliations:** 1 Centre for Cancer Biomarkers CCBIO, Department of Clinical Science, University of Bergen, Jonas Lies vei 65, N-5021, Bergen, Norway.; 2 University Hospital 12 de Octubre, Madrid, Spain; Lung Cancer Unit H12O-CNIO, Spain.; 3 Department of Chemistry and Centre for Pharmacy, University of Bergen, Allégaten 41, N-5007, Bergen, Norway.; 4 KinN Therapeutics AS, Jonas Lies vei 65, 5021 Bergen, Norway.; 5 Department of Pathology, Oslo University Hospital, Norwegian Radium Hospital, and Faculty of Medicine, Institute of Clinical Medicine, University of Oslo, Oslo, Norway.; 6 Molecular imaging Center, Department of Biomedicine, University of Bergen, Jonas Lies vei 91, N-5009, Bergen, Norway.; 7 Western Norway University College, Inndalsveien 28, N-5063, Bergen, Norway.; 8 Department of Pathology, Haukeland University Hospital, Jonas Lies vei 65, N-5021, Bergen, Norway.; 9 Centre for Pharmacy, Department of Clinical Science, University of Bergen, Jonas Lies vei 65, Bergen 5021, Norway.; 10 Vivarium, Department of Clinical Medicine, University of Bergen, Jonas Lies vei 65, 5021 Bergen, Norway.

**Keywords:** ^18^F-FMISO, Gene-directed enzymatic prodrug therapy, GDEPT, nitroreductase, NTR, cancer, xenograft, preclinical, mouse, PET/CT, imaging

## Abstract

Nitroreductases (NTR) are a family of bacterial enzymes used in gene directed enzyme prodrug therapy (GDEPT) that selectively activate prodrugs containing aromatic nitro groups to exert cytotoxic effects following gene transduction in tumours. The clinical development of NTR-based GDEPT has, in part, been hampered by the lack of translational imaging modalities to assess gene transduction and drug cytotoxicity, non-invasively. This study presents translational preclinical PET imaging to validate and report NTR activity using the clinically approved radiotracer,^ 18^F-FMISO, as substrate for the NTR enzyme.

**Methods:** The efficacy with which ^18^F-FMISO could be used to report NfsB NTR activity *in vivo* was investigated using the MDA-MB-231 mammary carcinoma xenograft model. For validation, subcutaneous xenografts of cells constitutively expressing NTR were imaged using ^18^F-FMISO PET/CT and fluorescence imaging with CytoCy5S, a validated fluorescent NTR substrate. Further, examination of the non-invasive functionality of ^18^F-FMISO PET/CT in reporting NfsB NTR activity *in vivo* was assessed in metastatic orthotopic NfsB NTR expressing xenografts and metastasis confirmed by bioluminescence imaging. ^18^F-FMISO biodistribution was acquired *ex vivo* by an automatic gamma counter measuring radiotracer retention to confirm *in vivo* results. To assess the functional imaging of NTR-based GDEPT with ^18^F-FMISO, PET/CT was performed to assess both gene transduction and cytotoxicity effects of prodrug therapy (CB1954) in subcutaneous models.

**Results:** ^18^F-FMISO retention was detected in NTR^+^ subcutaneous xenografts, displaying significantly higher PET contrast than NTR^-^ xenografts (*p* < 0.0001). Substantial ^18^F-FMISO retention was evident in metastases of orthotopic xenografts (*p* < 0.05). Accordingly, higher ^18^F-FMISO biodistribution was prevalent *ex vivo* in NTR^+^ xenografts. ^18^F-FMISO NfsB NTR PET/CT imaging proved useful for monitoring *in vivo* NTR transduction and the cytotoxic effect of prodrug therapy.

**Conclusions:** ^18^F-FMISO NfsB NTR PET/CT imaging offered significant contrast between NTR^+^ and NTR^-^ tumours and effective resolution of metastatic progression. Furthermore, ^18^F-FMISO NfsB NTR PET/CT imaging proved efficient in monitoring the two steps of GDEPT, *in vivo* NfsB NTR transduction and response to CB1954 prodrug therapy. These results support the repurposing of ^18^F-FMISO as a readily implementable PET imaging probe to be employed as companion diagnostic test for NTR-based GDEPT systems.

## Introduction

Gene directed enzyme prodrug therapy (GDEPT) is a promising anti-cancer strategy that aims to reduce off-target toxicity and limit severe side-effects by the combination of a prodrug and its activating enzyme 1. In a first step, tumours are transduced to express a gene encoding for the prodrug-activating enzyme. In a second step, a non-toxic prodrug is delivered systemically and subsequently converted *in situ* to a cytotoxic derivative by the expressed enzyme 2. In this way, GDEPT achieves high concentration of cytotoxic compounds locally, minimising systemic side effects associated with conventional cancer chemotherapy 3.

**Figure 1:**
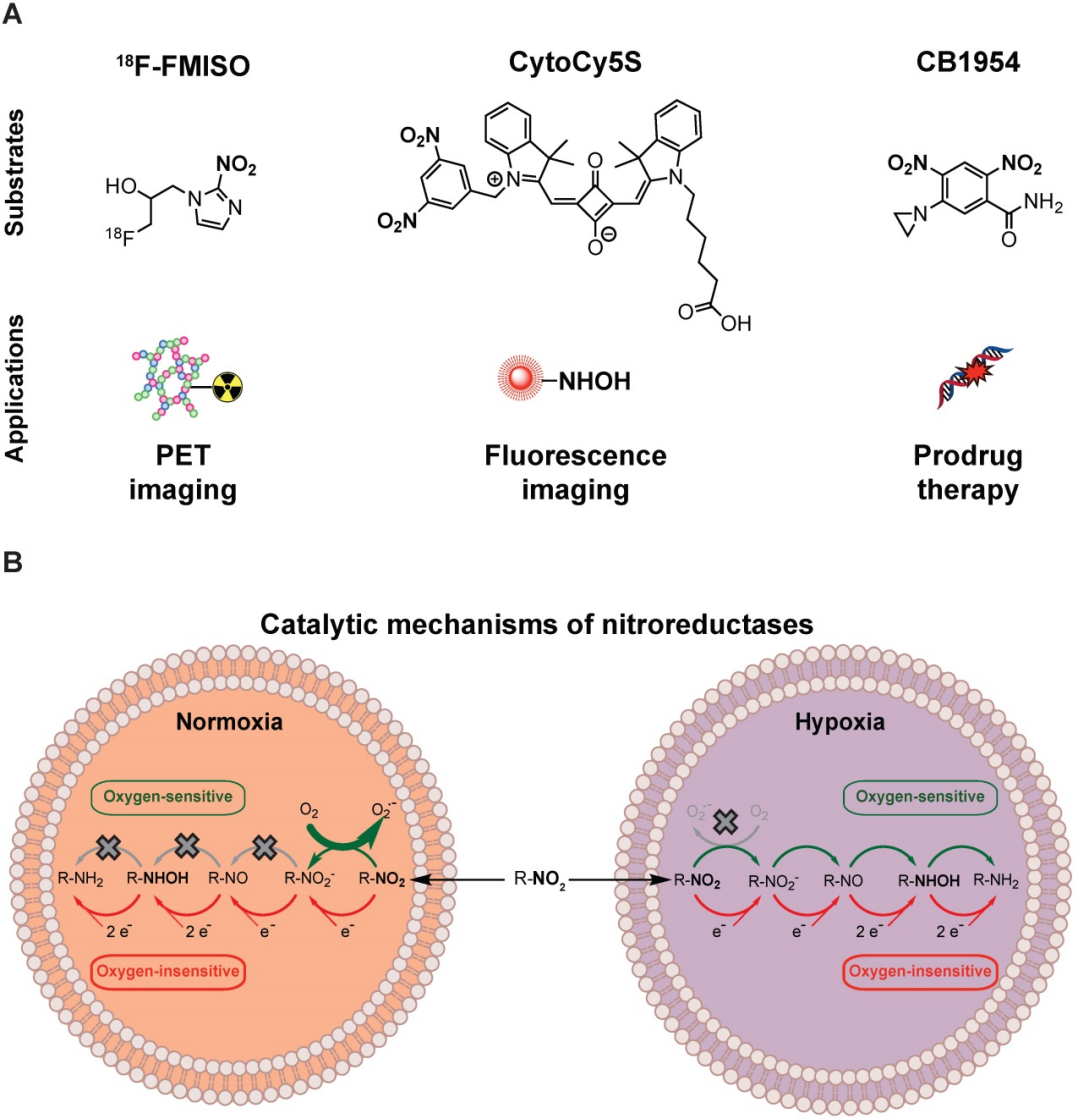
** Nitroreductases and nitroaromatic substrates for multiple applications.** (A) Nitroreductase subtrates employed in imaging or GDEPT. (B) Comparison of the catalytic mechanisms of oxygen-sensitive and oxygen-insensitive nitroreductases in normoxic and hypoxic conditions.

The most extensively studied prodrug/enzyme pairs for GDEPT are ganciclovir (GCV)/herpes simplex virus-thymidine kinase (HSV-TK) 4 and 5-(aziridin-1-yl)-2,4-dinitrobenzamide (CB1954, Figure 1B)/*E. Coli* nitroreductase NfsB (onwards referred as NTR) 5. HSV-TK phosphorylates GCV, allowing its incorporation into DNA, resulting in toxicity due to interference with the DNA synthesis 6. NTR reduces the nitro groups (R-NO_2_) present in CB1954, into hydroxylamines (R-NHOH), resulting in the formation of an alkylating agent with cytotoxic activity 7, 8. Moreover, the diffusion of the activated compound into neighbouring non-targeted cells amplifies this cytotoxic effect (bystander effect) 9, 10. Several trials have evaluated the clinical application of HSV-TK and NTR-based GDEPT strategies with contrasting results 5, 11, 12. The largest clinical trial, including 124 patients receiving HSV-TK-based GDEPT, reported no difference in comparison to standard therapy. Modest HSV-TK expression was suggested as a potential limiting factor 11. Indeed, a phase II HSV-TK-based GDEPT trial using a more efficient gene transduction strategy reported a significant increase in survival 12. These studies illustrate the need for monitoring the gene transduction efficiency. One of the NTR-based GDEPT clinical trials assessed the transduction efficiency using immunohistochemistry in tumour biopsies 5. However, immunohistochemistry is time consuming and may be affected by inadequate sampling of representative tumoral areas. For large clinical trials, non-invasive techniques such as imaging, which allows for easier clinical implementation, are required. In this respect, positron emission tomography (PET), which is routinely employed in clinical settings, enables detection of the activity for some of the enzymes used in the GDEPT strategies and may be suitable for interrogation of transduction efficiency in clinical trials 13, 14.

The activity of HSV-TK can be evaluated using radiolabeled probes, such as ^18^F-FIAU (1-(2- deoxy-2-[^18^F]-fluoro-1-D-arabinofuranoside)-5-iodouracil), ^18^F-FHBG (9-[4-[^18^F]fluoro-3-(hydroxymethyl) butyl]guanine) and GCV analogues 15, 16. HSV-TK PET/CT imaging has indeed been successfully applied for monitoring transduction efficiency, tissue specificity and therapeutic effect preclinically 15, 17. Subsequently, HSV-TK PET/CT imaging was incorporated into phase I clinical trials to monitor transduction efficiency in healthy individuals and in cancer patients 18, 19. In an ongoing phase I clinical trial investigating the use of HSV-TK-based GDEPT in combination with immunotherapy (NCT04313868), HSV-TK PET/CT imaging with ^18^F-FHBG has been implemented to explore the effect of viral administration routes in transduction efficacy.

NTR activity has been effectively imaged in a preclinical context using the near-infrared fluorescent probe CytoCy5S 20 and a caged bioluminescent substrate “NTR caged luciferin” (NCL) 21. However, the non-invasive clinical application of optical imaging approaches is challenging due to limited tissue penetrance of light and associated autofluorescence. This could potentially be overcome by employing PET as the imaging modality, provided that an appropriate NTR specific tracer is available. For this purpose, we have focused on radiolabelled 2-nitroimidazoles (2-NI) that have been extensively utilised as hypoxia imaging agents (e.g. FMISO or FETNIM) 22, 23. Hypoxia induces expression of oxygen-sensitive nitroreductases, able to convert 2-NI (e.g. FMISO, Figure 1A) into the corresponding hydroxylamines, which covalently bind to macromolecules in the tumour, allowing PET contrast 24. The mechanistic similarity between oxygen-sensitive and *E.coli* nitroreductases, oxygen-insensitive, (Figure 1B) and their ability to convert aromatic nitro groups of various substrates (Figure 1A) into aromatic hydroxylamines, render 2-NI as plausible probes for clinical imaging of NTR activity 25.

Recently, Mowday and co-workers 26 have demonstrated effective NTR PET imaging employing *NfsA* from *E. Coli* in combination with the 2-NI PET tracer ^18^F-HX4. Similarly, in this work we have investigated whether ^18^F-FMISO, a PET tracer approved by FDA as an investigational new drug for human use, could be repurposed as a PET probe for non-invasive imaging of NTR for application in the context of GDEPT. ^18^F-FMISO was validated as an NTR specific PET imaging tracer using mammary carcinoma xenograft models. Further, the applicability of ^18^F-FMISO imaging was demonstrated in the two steps of GDEPT i.e. gene transduction and cytotoxicity effects of prodrug therapy. These results support ^18^F-FMISO as a readily implementable NTR specific PET imaging probe to be employed as a companion diagnostic test for NTR-based GDEPT.

## Materials and methods

### *In vitro* experiments

#### Cell culture

Cells were maintained in a humidified atmosphere at 37 °C in 5% CO_2_ in complete medium, consisting of DMEM (Sigma-Aldrich, MO, USA) or RPMI-1640 supplemented with 10% FBS, 1% penicillin/streptomycin (Sigma-Aldrich), and 1% L-glutamine (Sigma-Aldrich). 293T cells were purchased from DSMZ (Braunschweig, Germany). MDA-MB-231^Luc+^ and NCI-H460^ Luc+^ cells, stably expressing luciferase, (onwards referred to as NTR^-^) were kindly provided by Prof. James Lorens (University of Bergen). Generation of MDA-MB-231^Luc+GFP+NTR+^ and NCI-H460^Luc+GFP+NTR+^ cells, stably expressing luciferase, GFP and NfsB (onwards referred to as NTR^+^) has been previously described by McCormack et al. 20. Characterisation of the cell lines by flow cytometry can be found in Figures S1 and S3. To avoid possible expression drift due to cell culturing, NTR^+^ cells were sorted prior to the experiments employing the flow cytometric methods described previously.

#### NTR lentivirus production

A custom-made lentiviral expression vector, pCDH-EF1α-NTR, containing the coding sequence of *E. coli NfsB* gene under the control of the EF1α promoter was produced by System Bioscience (CA, USA). Packaging into VSV-G pseudotyped viral particles was performed by co-transfection of pCDH-EF1α-NTR, pMD2.G and psPAX2 (Figure S1) into 293T cells. Viral titers were reported as transforming units (TU) per mL. TU were measured by transduction of 293T cells with serial dilutions of conditioned medium from virus packaging cells and analysis of NTR expression using a flow cytometric method described previously by McCormack et al. 20. For validation purposes, it was confirmed that NTR expression levels in MDA-MB-231 cells transduced with pCDH-EF1α-NTR and in the NTR^+^ cell line were within the same range (Figure S1).

#### Flow cytometry

For the analysis of NTR expression 0.1 x 10^6^ cells were incubated at 37 °C with DMEM supplemented with 1 µM of CytoCy5S for 1 hour. All samples were washed twice with PBS + 2% BSA before acquisition with the BD Accuri C6 flow cytometer (BD Biosciences, NJ, USA) with a laser excitation at 640 nm and emission filter at 675 ± 12.5 nm.

### *In vivo* experiments

#### General animal care

All experiments were approved by The Norwegian Animal Research Authority (FOTS approval ID 9059) and conducted according to The European Convention for the Protection of Vertebrates Used for Scientific Purposes. NOD-*scid* IL2Rg^null^ mice (referred to as NSG) were bred at Vivarium (University of Bergen). Mice were housed in groups of ≤ 5 in individually ventilated cages (Techniplast, Italy). General condition and body weight were recorded twice per week.

#### MDA-MB-231 and NCI-H460 subcutaneous xenografts

Tumours were engrafted subcutaneously mono- or bi-laterally in the scapular area. 5 × 10^6^ cells were prepared in 50 μL DMEM with 25% BD Matrigel^™^ (BD Science, CA, USA) and injected at each site to form a single tumour. Tumour volumes were measured weekly with a digital calliper and calculated using the ellipsoid volume formula: Volume = π (length × width × height)/6.

#### MDA-MB-231 orthotopic xenografts

1 × 10^6^ cells were prepared in 50 μL of DMEM with 25% BD Matrigel^™^ (BD Science) and engrafted in the right inguinal mammary fat pad, as previously described 20, 27. Prior to cell implantation, mice were injected with 0.1 mg/kg buprenorphine (Temgesic, Indivior, UK) for analgesia.

#### CB1954 treatment

CB1954 (Sigma-Aldrich) was resuspended in DMSO at 26.6 mg/mL. CB1954 treatment was initiated when mean tumour volumes reached 172 ± 65 mm^3^ in NTR^+^ and NTR^-^ xenografts or four weeks after *in vivo* transduction in NTR transduced xenografts. Animals received two intraperitoneal (i.p.) treatments in a six-day interval with a dose of 40 mg/kg diluted in saline.

#### *In vivo* NTR transduction with lentiviral particles

NTR lentiviral particles were concentrated using Lenti-Pac Lentivirus Concentration solution (Genecopoeia, MD, USA). Subcutaneous xenografts were transduced *in vivo* by intratumoral injection of 8 × 10^7^ TU resuspended in 75 μL of the complete medium supplemented with 5 μg/mL Polybrene (Sigma-Aldrich) when mean tumour volume was 70 ± 25 mm^3^. A single injection of viral particles per tumour was performed administering the viral suspension throughout the diameter of the tumour.

#### ^18^F-FMISO biodistribution

Tumours and organs were harvested from four mice, two hours post i.v. administration of ^18^F-FMISO (150 µL, 8-12 MBq, 228-342 MBq/kg) and radiation was measured on a Wizard2® Automatic Gamma Counter (Perkin Elmer, MA, USA). The results are expressed as a percentage of the injected dose per gram of tissue (% ID/g).

#### Histopathology examinations

Following euthanasia, the tumour and the organs were fixed in neutral buffered formalin solution 10% (Sigma-Aldrich) at room temperature for 24 hours and later kept in PBS and stored in darkness at 4 °C. 5 μm thick sections were stained with haematoxylin and eosin (HE) and were examined by a pathologist for verification of malignancy.

Immunohistochemical (IHC) staining was done on tumour tissue sectioned at 5 µm thickness. Deparaffinisation and antigen retrieval was performed with EnVision FLEX Target Retrieval Solution, Low pH (DAKO, Copenhagen, Denmark) on a Dako PT Link instrument (Dako). After antigen retrieval, tissues were incubated for 8 min with peroxidase blocker (Peroxidase Blocking Reagent, cat #S2001, Agilent Technologies) and thereafter for 10 min with protein blocker (Protein Block Serum-free, cat # X090930-2, DAKO) at room temperature. The blocking solution was then removed and the slide was wiped dry around the tissue section before application of the primary antibody. The sections were then incubated overnight at 4 ºC degrees with the HIF1α primary antibody (ab51608, Abcam, Cambridge, UK) dilution 1:300. The staining was performed on a DAKO Autostainer using the EnVision+ System-HRP Labelled Polymer Anti-Rabbit K4002 as secondary antibody for 30 min (Agilent Technologies, Norway). 3,3`-Diaminobenzine (DAB+) Substrate-Chromogen was used as chromogen for 10 min. Sections were counterstained with haematoxylin (cat # S3301, Agilent Technologies) for 10 min, dehydrated, and mounted with a coverslip (Agilent Technologies) using Pertexx mounting medium (Histolab Products AB, Askim, Sweden). Human placenta with known reactivity to the selected marker was used as positive control.

Whole tissues were scanned employing an Olympus VS120 S6 Slide scanner (Olympus Corporation, Tokio, Japan). Quantification of the normalised HIF1α positive area was performed employing FIJI 28, following the protocol described by Crowe et al. 29.

#### PCR for monitoring *in vivo* NTR transduction

Following euthanasia, part of the tumour was snap-frozen on dry ice and stored at -80 ºC. DNA was purified using QIAamp DNA Mini Kit (QIAGEN, Germany). A 348 bp fragment of the coding sequence of *E. coli NfsB* gene was PCR amplified in a thermocycler under standard conditions using *Taq* DNA Polymerase (Invitrogen, CA, USA) and the following primers: NTR-F 5' GCGTCATTCCACTAAGGCAT 3' and NTR-R 5' GCGAAGAACTTGCGACCTTT 3'. DNA amplification was visualised by gel electrophoresis followed by imaging on a Gel Doc EZ system (Bio-Rad, CA, USA). Nancy-520 (Sigma-Aldrich) was used as an intercalating agent and 1 kb DNA Ladder (Sigma-Aldrich) to estimate DNA size.

### Imaging techniques

During bioluminescence and fluorescence imaging mice were anaesthetised with 1.5% isoflurane (Abbot Labor Ltd, IL, USA), 3-4% sevoflurane (Abbot Labor Ltd) during PET/CT imaging.

### Bioluminescence imaging

D-Luciferin (Biosynth, Switzerland) was administered i.p. at a dose of 150 mg/kg 10 minutes before imaging. Images were acquired using an In-Vivo FX Pro molecular imaging system (Carestream Health Inc., NY, USA). Analysis was performed with the Carestream molecular imaging software v5.0.6.20.

#### CytoCy5S-Fluorescence imaging

CytoCy5S was synthesised following a published method 20. 100 µL of a 1 mM CytoCy5S solution were injected intravenously. Imaging was performed after a 1-hour washout using an IVIS Spectrum imaging system (PerkinElmer, MA, USA.). Analysis was performed with the Living Imaging® software v4.5 (PerkinElmer).

### ^18^F-FMISO PET/CT imaging

#### ^18^F-FMISO synthesis

^18^F-fluoride was produced by the ^18^O(p,n)^18^F-reaction in a niobium target on a GE PETtrace6 cyclotron (GE Healthcare,) with EOB-activities in the range of 80 - 100 GBq. The activity was delivered to the synthesis hot-cell by a Safe Transfer System (Skistad Elektroautomasjon AS, Norway). ^18^F-FMISO was produced on a FASTlab2 (GE Healthcare, IL, USA) with the synthesis sequence provided by the supplier. The radiosynthesis has been described in detail previously 30, 31. Reagents and synthesis cassette were purchased from GE Healthcare Norway AS (Norway). Purification of ^18^F-FMISO was performed through a series of SPE-cartridges, and thus did not require any semi-preparative HPLC. The final ^18^F-FMISO formulation contained approx. 7% ethanol. Total synthesis time was approximately 50 minutes and yields in the order of 20-30% (not decay corrected).

#### PET/CT imaging

PET/CT scans were acquired using the integrated nanoScan PC PET/CT (Mediso Ltd, Hungary) featuring spatial resolution of 800 μm and 300 μm of the PET and CT detector systems, respectively. The field of view (FOV) was 9.6 × 10 cm in axial direction and transaxial direction allowing whole-body imaging of the mice. The PET detectors consist of LYSO crystals, and acquisition was performed in a 1:5 coincidence and normal count mode. Mice were scanned using a dual mouse bed with integrated heating (37 °C). Each PET scan was conducted over 30 minutes, 1.5 hours post i.v. administration of ^18^F-FMISO (8-12 MBq, 228-342 MBq/kg). Prior to PET acquisition, a whole-body CT scan (helical projections with tube energy of 50kVp, exposure time 300 ms, 720 projections, max FOV, binning 1:4) was performed providing anatomical information, as well as attenuation correction PET image reconstruction.

#### PET/CT reconstruction and processing

PET images were reconstructed using the Nucline software by employing the Tera-Tomo 3D (OSEM) algorithm (four iterations and six subsets, 1-5 coincidence mode) and the following corrections: depth-of-interaction (DOI), randoms, crystal dead time, normalisation. Prior to PET, a whole-body CT (low energy) was acquired for anatomic reference and attenuation correction. CT images were reconstructed using a RamLak filter. The PET and CT images were co-registered automatically. Images were reconstructed with a voxel size of 0.25×0.25×0.25 mm^3^ for CT, and 0.4×0.4×0.4 mm^3^ for PET. Data analyses were performed using InterView Fusion version 3.03.078.0000 (Mediso Ldt.). Standard uptake value (SUV) was calculated using the equation: SUV = C_PET_(T)/(ID/BW), where C_PET_(T) was the measured activity in tissue, ID the injected dose measured in kBq, and BW the mouse's body weight in kg. For each scan a spherical volume of interest (VOI) with radius 2 mm was drawn manually over the muscle in the neck and SUV_mean_ was calculated to serve as reference. This value enabled segmentation of putative tumour tissue having SUV ratios of twice higher than reference for ^18^F-FMISO. VOIs of primary tumours and of likely metastases were drawn semi-automatically in the PET images for estimation and calculation of SUV_max_ and SUV_peak_ values. SUV_max_ is the maximum SUV value of all voxels included in the VOI. For SUV_peak_ analysis a specific volume was set (5 mm^3^ for SUV_p5_ and 10 mm^3^ for SUV_p10_) and all the possible spheres with that volume fitting inside the VOI were identified. The average SUV of all voxels within the spheres was calculated for all possible spheres and the highest average SUV values from this analysis were reported as SUV_p5_ and SUV_p10_. The SUV_peak_ analyses were performed using PMOD software (Version 3.8).

### Statistics

Results are given as mean ± standard deviation (SD). All statistical tests were performed using GraphPad Prism v 6.0h (GraphPad Software Inc, CA, USA) and *p* < 0.05 was considered significant. After randomisation, a one-way ANOVA was applied to ensure unbiased assignment of tumour volumes among the experimental groups. Comparison of means was performed using Student's t-tests. Test for equality of variances was performed using an F test. When variances were not equal Welch's correction was applied to Student's t-tests. Correlations were analysed by computing Pearson's correlation coefficients.

## Results

### ^18^F-FMISO permits imaging of NTR expression in subcutaneous xenograft models

Although fluorescent 20 and chemiluminescent 21 substrates have been successfully applied to report NTR expression in preclinical models of cancers, their clinical potential is limited. We hypothesised that ^18^F-FMISO, a clinically approved PET tracer known to be sequestered and reduced in hypoxic cancer environments, may provide a PET-based imaging modality for the visualisation of NTR activity *in vivo*. To examine the potential of ^18^F-FMISO as a PET tracer of NTR expression *in vivo*, ^18^F-FMISO NTR PET/CT imaging and CytoCy5S-FLI were compared in a subcutaneous xenograft model.

**Figure 2:**
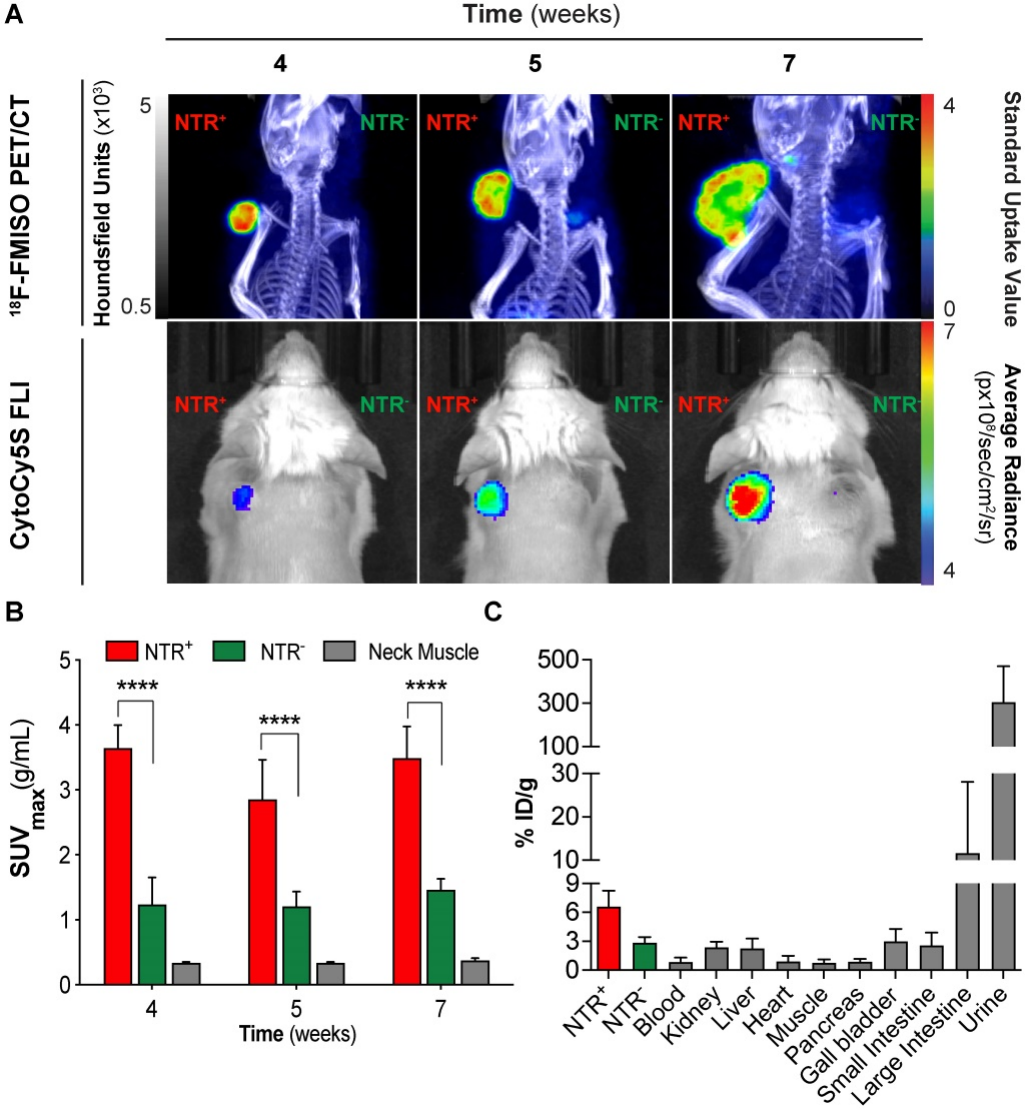
**^18^F-FMISO permits imaging of NTR expression in subcutaneous xenograft models.** (A) Representative ^18^F-FMISO PET/CT MIP images and CytoCy5S-FLI 2D surface weighted images over four weeks. NTR^-^ tumour (right flank) and NTR^+^ tumour (left flank). (B) SUV_max_ values, were significantly higher in NTR^+^ xenografts for all the time points (****, *p* < 0.0001, n=7). (C) *Ex vivo* biodistribution (n = 3) of ^18^F-FMISO obtained at week seven. Biodistribution observed mainly in large intestines and bladder. Biodistribution was higher in NTR^+^ tumours than in NTR^-^ tumours and remaining organs. The p-values are represented as indicated: * *p* < 0.05, ** *p* < 0.01, *** *p* < 0.001 and **** *p* < 0.0001.

Briefly, mice were xenografted bi-laterally in the scapular region with either NTR^+^ or NTR^-^ cells (n = 3 mice per group) in addition to one mouse implanted with both NTR^+^ and NTR^-^ on adjacent scapulae. Mice were imaged at three timepoints post-engraftment (weeks four, five and seven) and imaging results compared (Figure 2A). CytoCy5S-FLI confirmed significantly higher contrast between NTR^+^ and NTR^-^ xenografts from week five (*p* < 0.05, Figure 2A), when mean tumour volumes reached 243.6 ± 94.6 mm^3^. ^18^F-FMISO NTR PET/CT resulted in significantly higher contrast between NTR^+^ and NTR^-^ xenografts earlier, at week four, when mean tumour volumes were 154.0 ± 75.8 mm^3^. At week four, NTR^+^ and NTR^-^ xenograft SUV_max_ values were 3.64 ± 0.36 g/mL and 1.24 ± 0.42 g/mL respectively (*p* < 0.0001). Contrast remained significantly higher at weeks five and seven (Figure 2A and 2B). To confirm these results, ^18^F-FMISO tracer biodistribution was analysed *ex vivo.* The highest tracer retention was detected in the bladder, consistent with high renal clearance, and in the large intestine (Figure 2C), which is expected as a consequence of the expression of nitroreductases by the local microbiota. ^18^F-FMISO retention was higher in NTR^+^ compared to NTR^-^ xenografts and remaining organs. Thus, our results suggest that ^18^F-FMISO can act as an NTR PET tracer.

To confirm that the higher retention of ^18^F-FMISO observed in NTR^+^ is specific for NTR expression, we wanted to compare the hypoxic status of the NTR^-^ and NTR^+^ xenografts. The hypoxic status of the subcutaneous MDA-MB-231 NTR^-^ and NTR^+^ xenografts was assessed employing IHC to detect HIF1α accumulation (Figure S2A). Quantification of the normalised area positive for HIF1α shows no statistical significance between MDA-MB-231 NTR^-^ and NTR^+^ xenografts (Figure S2B). This result suggests that ^18^F-FMISO retention in NTR^+^ xenografts is not related to increased levels of hypoxia, confirming ^18^F-FMISO as an NTR PET tracer.

Next, we wanted to analyse the performance of ^18^F-FMISO as an NTR PET tracer in the presence of other nitroreductases, both oxygen-insensitive and oxygen-sensitive. For this purpose we selected the non-small cell lung carcinoma NCI-H460 cell line, with high endogenous levels of the nitroreductase DT-diaphorase 32 and increased levels of hypoxia in comparison to MDA-MB-231 (hypoxic fractions of 13.6% and 4.7% respectively 33, 34). First we confirmed *in vitro* that NTR^+^/NTR^-^ ratio was notably lower for NCI-H460 than for MDA-MB-231 cell line, being respectively 5.4 and 50 (Figure S3A). Then, mice were xenografted bi-laterally in the scapular region with either NTR^+^ or NTR^-^ NCI-H460 cells (n = 4 tumours per group). Mice were imaged two weeks after engraftment and imaging results compared (Figure S3B). ^18^F-FMISO NTR PET/CT resulted in significantly higher contrast between NCI-H460 NTR^+^ and NTR^-^ xenografts with SUV_max_ values of 2.07 ± 0.36 g/mL and 1.51 ± 0.30 g/mL respectively (*p* < 0.05; Figure S3C). Interestingly, no significant differences were observed between SUV_max_ values from MDA-MB-231 NTR^-^ xenografts (1.24 ± 0.42 g/mL) and from NCI-H460 NTR^-^ xenografts. These results suggest that ^18^F-FMISO can act as an NTR PET tracer, even in models with notable presence of oxygen-insensitive and oxygen-sensitive nitroreductases.

### ^18^F-FMISO imaging of NTR^+^ metastatic lesions in orthotopic xenograft models

**Figure 3:**
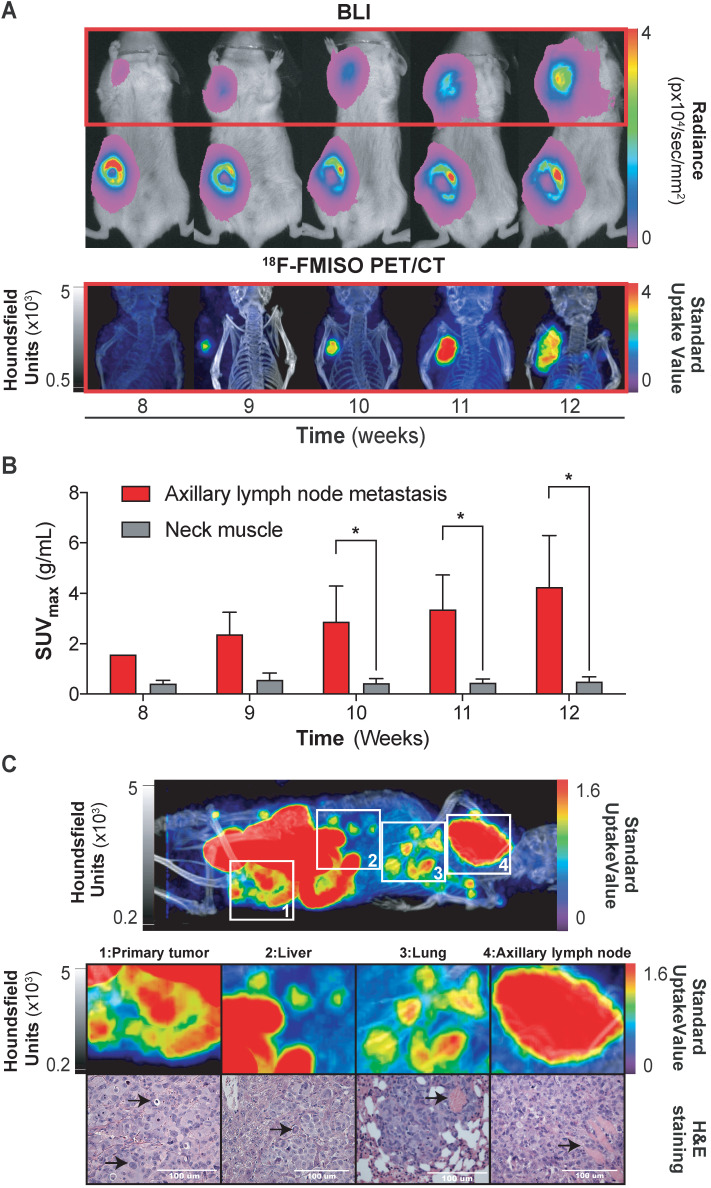
**^18^F-FMISO imaging of NTR^+^ metastatic lesions in orthotopic xenograft models.** (A) Representative images from week eight to 12. Presence of primary tumour (inguinal area) and axillary metastases (red square) by BLI. ^18^F-FMISO NTR PET/CT imaging in the axillary area. (B) Evolution of lymph node metastasis SUV_max_ compared to the reference tissue. Significant difference is observed at weeks ≥ 10 (*, *p* < 0.05), n = 3. (C) Whole body MIP images of ^18^F-FMISO NTR PET/CT show contrast in the primary tumour, liver, lung and axillary lymph node. HE staining shows widespread metastatic neoplasia in all the analysed organs. The histological features associated with neoplasia (marked with black arrows) include atypical mitosis, pyknosis, hyperchromatism, desmoplasia, irregular shape and size. Excessive extranodal tumour extension invading the adjacent muscle tissue can be seen in the axillary lymph (marked with black arrow). The lungs show signs of scattered micrometastases and venous infiltration (marked with black arrow). The p-values are represented as indicated: * *p* < 0.05, ** *p* < 0.01, *** *p* < 0.001 and **** *p* < 0.0001.

Having demonstrated the ability of ^18^F-FMISO to act as an NTR PET tracer in subcutaneous tumours, we next examined the suitability of the tracer for the detection of small metastatic lesions. We have previously visualised the axillary lymph node metastatic capacity of MDA-MB-231 cells when implanted orthotopically in immunodeficient mice *via* BLI 27. Thus, 1 × 10^6^ NTR^+^ cells were xenografted orthotopically in the mammary fat pad (n = 3) and imaged weekly with BLI and, from the initiation of metastasis, also with ^18^F-FMISO NTR PET/CT for five weeks (Figure 3A). Axillary lymph node metastases were clearly visible from week 8, with progressive increase in BLI up to week 12 (Figure 3A). Comparative ^18^F-FMISO NTR PET/CT imaging of the same mice demonstrated contrast at the axillary lymph node metastatic site with SUV_max_ values of 2.37 ± 0.88 g/mL from week 9 (Figure 3B). Metastatic contrast increased in weeks 10, 11 and 12 with SUV_max_ values of 2.88 ± 1.41, 3.36 ± 1.37 and 4.25 ± 2.04 g/mL, which were significant from background (SUV_max_ of reference tissue range: 0.4-0.56 g/mL; *p* < 0.05). Further analysis of ^18^F-FMISO NTR PET/CT images of NTR^+^ xenografts revealed additional contrast in the thoracic and lumbar regions of metastatic mice suggestive of hepatic and pulmonary metastasis (Figure 3C, upper). Subsequently, mice were euthanised, and suspected metastatic organs excised. Histological examination of HE stained sections of the lungs, liver and axillary lymph node demonstrated the presence of metastases in organs where accumulation of ^18^F-FMISO had been observed by ^18^F-FMISO NTR PET/CT imaging (Figure 3C, lower).

### ^18^F-FMISO allows assessment of NTR expression after *in vivo* transduction

**Figure 4:**
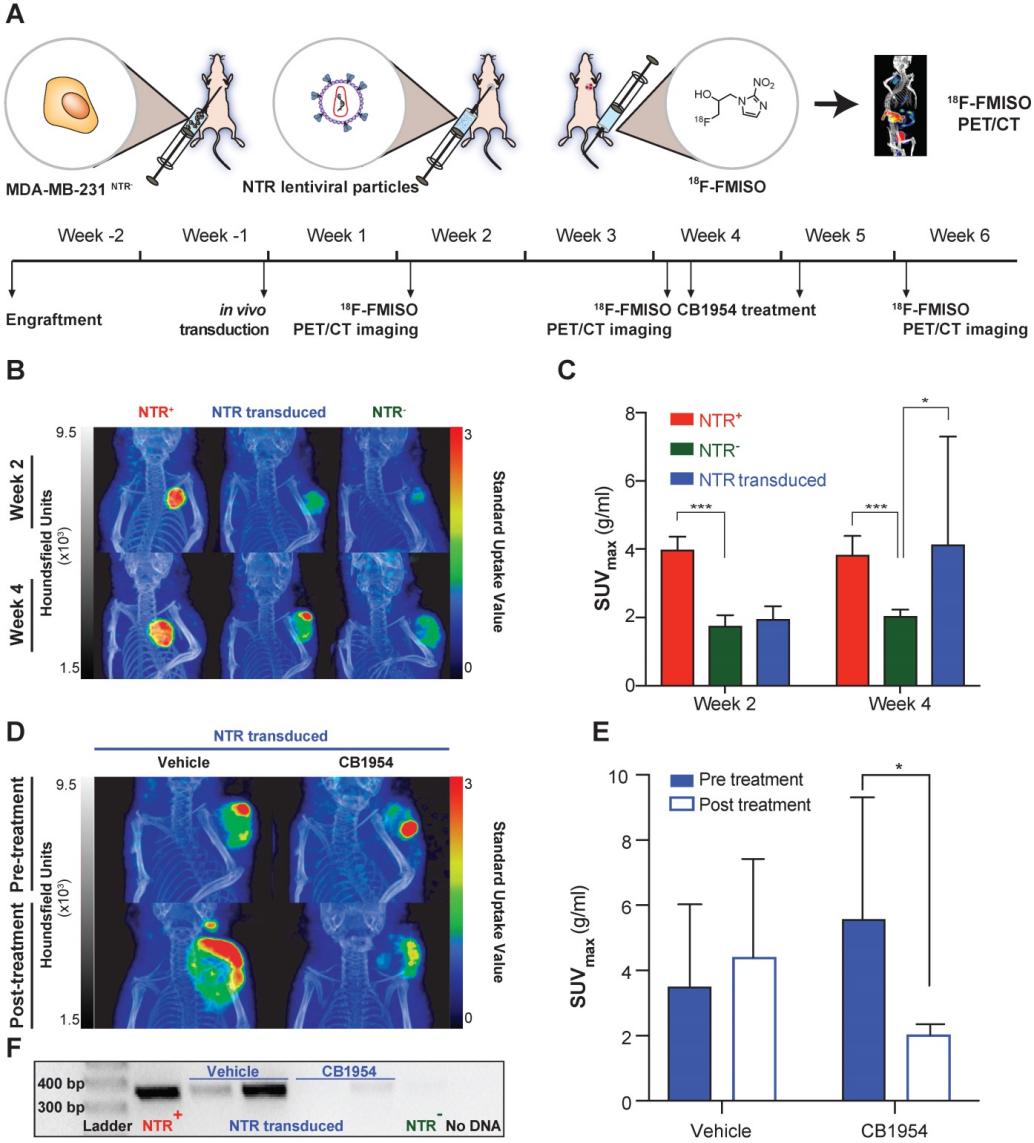
**^18^F-FMISO allows assessment of NTR expression after *in vivo* transduction.** (A) Experimental set-up for *in vivo* NTR lentiviral transduction, CB1954 treatment and ^18^F-FMISO PET/CT imaging. (B) Representative ^18^F-FMISO PET/CT MIP images suggesting the expression of NTR in the *in vivo* transduced tumours both at week two and four after transduction. (C) Four weeks after transduction, the SUV_max_ in the *in vivo* transduced tumours was significantly higher (*, *p* < 0.05 and ***, *p* < 0.001), n = 10). (D) Representative ^18^F-FMISO PET/CT MIP images suggesting a decrease in ^18^F-FMISO PET contrast in NTR transduced xenografts treated with CB1954 (E) SUV_max_ of the *in vivo* transduced tumours treated with vehicle showed a non-significant increase. SUV_max_ of the *in vivo* transduced tumours treated with CB1954 decreased significantly (*, *p* < 0.05 n = 6) (F) Confirmation of NTR transduction by PCR amplification of NTR coding sequences only in NTR^+^ xenografts and NTR transduced xenografts treated with vehicle. The p-values are represented as indicated: * *p* < 0.05, ** *p* < 0.01, *** *p* < 0.001 and **** *p* < 0.0001.

While the aforementioned ^18^F-FMISO imaging results demonstrate the useful application of ^18^F-FMISO NTR PET/CT imaging for detection of constitutive NTR expression, the imaging demands of GDEPT are far greater. Initially, imaging of GDEPT should permit visual confirmation of* in vivo* viral transduction, which will occur in a discrete fraction of the tumoral cells 11, 12. To examine the potential of ^18^F-FMISO NTR PET/CT imaging to assess *in vivo* transduction of tumour cells by NTR, an NTR lentiviral construct was designed (Figure S1). Viral particles were collected, concentrated and 8 x 10^7^ TU injected intratumorally into NTR^-^ subcutaneous xenografts (Figure 4A).

NTR transduced xenografts (n = 10), in addition to NTR^+^ and NTR^-^ controls (n = 4 per group) were ^18^F-FMISO NTR PET/CT imaged, two and four weeks post-transduction (Figure 4A). Clear contrast of NTR^+^ controls could be demonstrated over NTR^-^ controls both at week 2 and 4 (SUV_max_ of 3.97 ± 0.40 and 3.82 ± 0.57 g/mL respectively;* p* <0.001 Figure 4B & C). At week 2, minimal contrast was visualised in NTR transduced xenografts, albeit non-significant compared to NTR^-^ controls (SUV_max_ of 1.94 ± 0.38 and 1.74 ± 0.32 g/mL respectively; Figure 4C). Imaging at week 4 demonstrated significant contrast compared with NTR^-^ controls (SUV_max_ of 4.12 ± 3.18 and 2.03 ± 0.20 g/mL respectively; *p* < 0.05). Similar results were obtained when SUV_peak_ was analysed (Figure S4). Interestingly, contrast in lentiviral transduced tumours was observed at discrete foci (Figure 4B).

At week 4, NTR transduced xenografts were randomised to receive CB1954 (two 40 mg/kg i.p. injections in a six-day interval) or vehicle (Figure 4A). Post-treatment ^18^F-FMISO NTR PET/CT imaging, at week 6, showed a further increase in contrast in xenografts treated with vehicle, although non-significant (pre-treatment SUV_max_ of 3.5 ± 2.5 and post-treatment SUV_max_ of 4.4 ± 3.0 g/mL Figure 4D & E). On the other hand, a significant decrease in contrast was observed in xenografts treated with CB1954 (pre-treatment SUV_max_ of 5.57 ± 3.73 and post-treatment SUV_max_ of 2.02 ± 0.32 g/mL; *p* < 0.05; Figure 4D & E).

To confirm these imaging results, tumours were isolated post necropsy, DNA isolated and NTR transduction was corroborated by PCR amplification of an NTR coding sequence in the NTR transduced xenografts treated with vehicle and NTR^+^ controls (Figure 4F). These results confirm the utility of ^18^F-FMISO NTR PET/CT imaging for detecting NTR expression after* in vivo* viral transduction.

To exclude any possible confounding effect on ^18^F-FMISO NTR PET/CT imaging arising from hypoxia induced by intratumoral injection, at the end of the experiment the accumulation of HIF1α was measured in NTR^-^ transduced xenografts and compared with NTR^-^ tumours not receiving an intratumoral injection. The spatial pattern of HIF1α accumulation was similar in both tumour groups (Figure S5A) and the mean normalised HIF1α positive area was indeed significantly lower in intratumorally injected tumours (Figure S5B). All the above-mentioned data confirms ^18^F-FMISO NTR PET/CT imaging as a technique able to detect *in vivo* NTR viral transduction.

### ^18^F-FMISO NTR PET/CT imaging reports CB1954 treatment efficacy in NTR expressing xenografts

**Figure 5:**
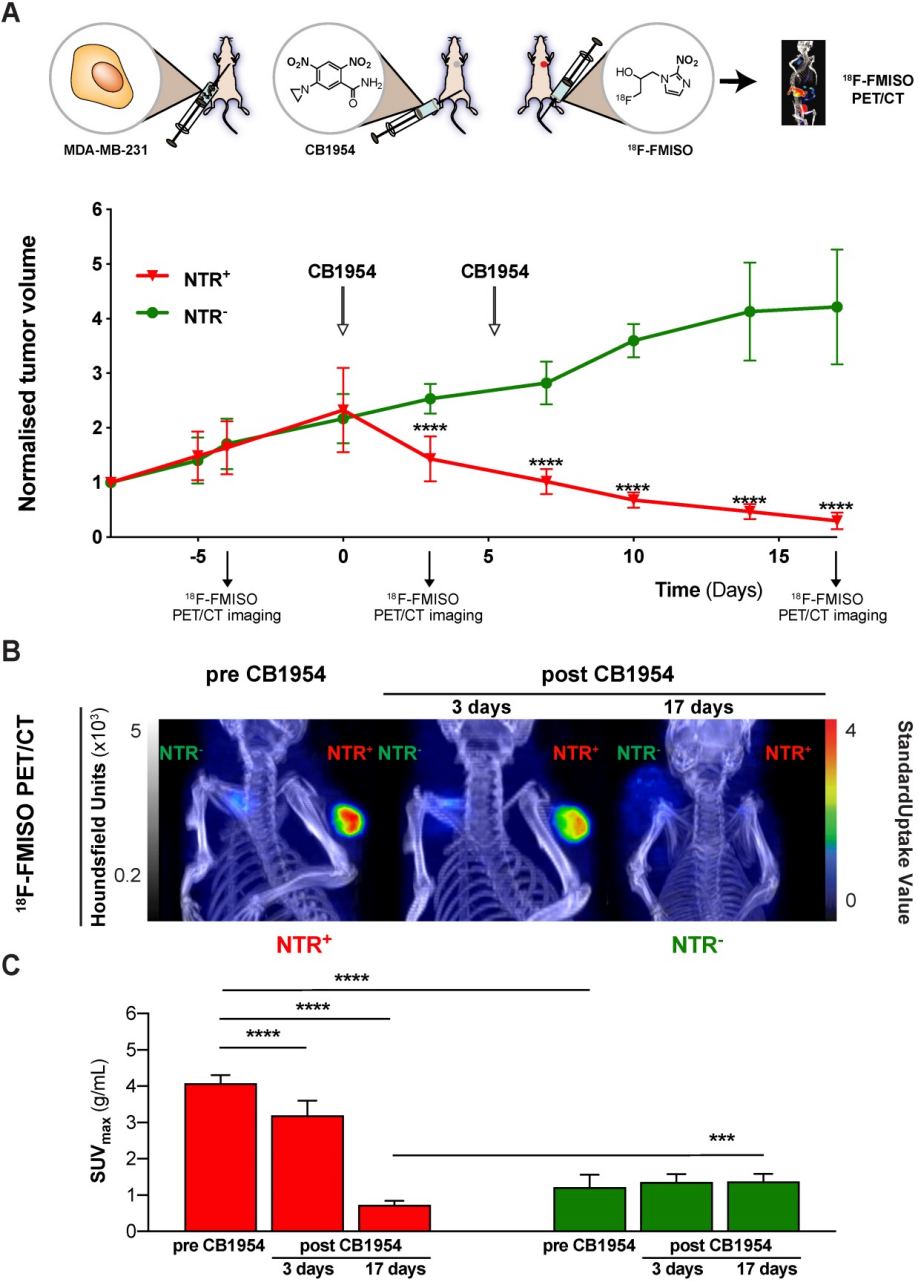
**^18^F-FMISO NTR PET/CT imaging reports CB1954 treatment efficacy in NTR expressing xenografts.** (A) CB1954 treatment response and experimental set-up for treatment monitoring employing ^18^F-FMISO PET/CT imaging (B) Representative ^18^F-FMISO PET/CT MIP images from NTR^-^ tumour (left flank) and NTR^+^ tumour (right flank) before treatment and three and 17 days after CB1954 dosing. (C) Before treatment, the SUV_max_ values were significantly higher in NTR^+^ than in NTR^-^ tumours (****, *p* < 0.0001, n = 7). Three and 17 days after, the SUV_max_ became significantly lower in NTR^+^ xenografts in comparison to before treatment (****, *p* < 0.0001, n = 7). After 17 days, SUV_max_ of NTR^+^ xenografts became significantly lower than the corresponding SUV_max_ of NTR^-^ xenografts (***, *p* < 0.0001, n = 7). The p-values are represented as indicated: * *p* < 0.05, ** *p* < 0.01, *** *p* < 0.001 and **** *p* < 0.0001.

Having demonstrated the potential of ^18^F-FMISO NTR PET/CT imaging to successfully visualise *in vivo* NTR transduction, the capacity of ^18^F-FMISO NTR PET/CT imaging to report on CB1954 prodrug treatment efficacy was examined. NTR^-^ and NTR^+^ subcutaneous xenografts (n = 7 per group) were treated with CB1954 (two 40 mg/kg i.p. injections in a six-day interval) (Figure 5). ^18^F-FMISO NTR PET/CT imaging was performed pre-treatment (day -4), during treatment (day 3) and following completion of treatment (day 17), in addition to calliper measurements of tumour volumes (Figure 5A). As expected, pre-treatment ^18^F-FMISO NTR PET/CT imaging of NTR^+^ xenografts resulted in significant contrast in comparison to NTR^-^ xenografts (SUV_max_ of 4.07 ± 0.23 and 1.22 ± 0.35 g/mL respectively; *p* < 0.001 (Figure 5B and C). At day 3, a significant decrease in NTR^+^ tumour volumes was observed versus corresponding NTR^-^ xenografts (normalised tumour volumes 2.53 ± 0.27 and 1.43 ± 0.41 mm^3^; *p* < 0.0001). Similarly, significant decrease in NTR^+^ tumour SUV_max_ values was also noted in ^18^F-FMISO PET contrast performed at day 3 in comparison to pre-treatment SUV_max_ values (SUV_max_ 4.07 ± 0.23 and 3.19 ± 0.41 g/mL; *p* < 0.001). Progressive reductions in NTR^+^ xenograft tumour volume continued following the completion of CB1954 treatment at days 7, 10 and 14 (*p* < 0.001), with normalised volumes of 0.1 ± 0.02 at termination of the experiment (Figure 5A). Final PET imaging of xenografts on day 17 (Figure 5B) confirmed calliper measurement results, with observed SUV_max_ values reduced to 0.72 ± 0.12 g/mL (*p* < 0.0001) (Figure 5C). These preclinical results validate translational development of ^18^F-FMISO PET/CT for prodrug treatment efficacy monitoring in the context of NTR-based GDEPT.

## Discussion

GDEPT has been suggested as an attractive novel therapeutic strategy aiming to address both drug resistance and off-target cytotoxicity 35, with promising preclinical results 36-39 and a number of HSV-TK-based GDEPT phase II/III clinical trials opened in the last five years (NCT02831933; NCT02768363; NCT03004183; NCT03541928; NCT03603405; NCT03596086; NCT02446093). Recent phase I studies (NCT03281382; NCT04313868) are evaluating the use of HSV-TK-based PET imaging as a companion diagnostic tool, facilitating the analysis of HSV-TK transduction efficacy and prodrug therapy response.

In the current study, we have investigated the repurposing of ^18^F-FMISO, an FDA approved PET tracer as investigational new drug for human use, as a companion diagnostic tool for NTR-based GDEPT. Employing a mammary carcinoma xenograft model constitutively expressing NTR, we demonstrated that ^18^F-FMISO PET/CT imaging is sensitive for detection of NTR, even in small metastases. ^18^F-FMISO NTR PET/CT imaging was found to be effective for detection of *in vivo* NTR transduction and for monitoring response to CB1954 treatment, demonstrating the feasibility of ^18^F-FMISO NTR PET/CT imaging in the context of GDEPT.

Whilst this work has utilised breast and lung cancer models, ^18^F-FMISO NTR PET/CT imaging is expected to be applicable to any solid tumour type. As suggested by our results, the interaction between ^18^F-FMISO NTR and ^18^F-FMISO hypoxia PET/CT imaging does not appear to compromise this NTR imaging strategy. Indeed, preclinical ^18^F-FMISO hypoxia PET/CT imaging in many different tumour types reported SUV_max_ values in the range of 0.19-0.70 mg/mL 40-45, notably lower than the SUV_max_ values values for ^18^F-FMISO NTR PET/CT imaging reported in this study (1.5-9.95 mg/mL).

The general applicability of ^18^F-FMISO NTR PET/CT imaging might be limited by the distribution of the tracer to the bladder and large intestine 46, which might interfere with the imaging of tumours in the abdominal cavity. One strategy to reduce PET signal in the abdominal cavity is to increase the bowel motility using laxatives prior to imaging, this has been explored in preclinical models 47. Another possible solution would be to employ *NfsA*, which is an NTR orthologue with higher affinity towards ^18^F-FMISO 26. *NfsA* may permit the use of lower ^18^F-FMISO doses, resulting in a decrease in the retention in the abdominal cavity.

We acknowledge the limitations and sub-optimal PK properties of ^18^F-FMISO. However, the extensive clinical use of this tracer for PET imaging of hypoxia makes the process of approval for a repurposed application easier. In parallel, novel tracers with optimised pharmacokinetic properties, in order to achieve high tumour accumulation and rapid healthy tissue clearance, are under development. A second generation 2-NI, ^18^F-DiFA, has displayed slightly improved hypoxia sensitivity in clinical studies, accompanied by minimal distribution into the gastrointestinal tract 46, 48 and is an interesting candidate for further studies in the context of NTR imaging.

In comparison to HSV-TK-based GDEPT, NTR-based GDEPT possesses higher therapeutic potential, as NTR-based GDEPT targets cells independently of the cell cycle phase or proliferation status 49 and it results in a notable bystander effect caused by the diffusion of activated derivatives through the cell membrane 9, 10. Despite the superior therapeutic potential of NTR-based GDEPT, clinical development is still in the early phases, as several of the steps for the NTR-based GDEPT strategy still need optimisation.

Efforts for optimisation of NTR-based GDEPT have in recent years focused on the key aspects of the technology, namely the delivery system, the NTR-enzyme and the prodrug. In an NTR GDEPT clinical trial, immune responses to the viral particles were detected 5. Although it is not known if immune mediated responses will compromise the therapeutic efficacy, new gene delivery systems amenable for clinical translation, such as extracellular vesicles 50 and ultrasound and microbubble mediated sonoporation have been investigated 51. While *NfsB* from *E. coli* has been the most commonly employed gene for NTR-based GDEPT, different NTR orthologs and homologs, with a much higher catalytic activity, such as *NfsA* from *E. coli* and *Nme* from *N. meningitidis* have been evaluated 26, 52, 53. Finally, development of improved prodrug candidates is ongoing 54, since CB1954 has been associated with dose-limiting hepatotoxicity in humans 55. Indeed, the last two aspects, NTR-enzyme and prodrug, have been optimised simultaneously through the engineering of a modified NTR enzyme that confers higher catalytic activity for an improved prodrug. Remarkable efforts by Gungor and co-workers employing an NTR from *S. saprophyticus, Ssap*-NtrB, and a library of different nitro-containing scaffolds has led to promising *in vitro* prodrug candidates, such as benzamide derivatives *N*-(2,4-dinitrophenyl)-4-nitrobenzamide, A5 or A20, and piperazine derivatives NHN12 or NHN14 54, 56-58. Other examples of such a strategy include *HChrR6* with CNOB 59, 60 and *NfsA* variant no. 22 with PR-104A 61. Interestingly, *NfsA* variant no. 22 retained reductive activity towards the hypoxia PET tracers, ^18^F-EF5 and ^18^F-HX4, allowing the possibility of NTR PET/CT imaging as companion diagnostic tool, as shown in the recently published work of Mowday et al. 26, 61.

Increasing preclinical knowledge will contribute significantly to the successful clinical translation of NTR-based GDEPT. The combination of a clinically amenable gene delivery system, optimised NTR enzymes and improved prodrugs is expected to boost the clinical application of NTR-based GDEPT.^ 18^F-FMISO NTR PET/CT imaging should be integrated into the clinical testing of future NTR-based GDEPT strategies, providing a robust and sensitive technology to monitor forthcoming clinical trials. ^18^F-FMISO NTR PET/CT is an FDA methodology readily available for clinical application. Our preclinical studies further establish this strategy as a strong candidate for companion diagnostic testing of NTR-based GEDPT.

## Supplementary Material

Supplementary figures.
