# Supplementary material for: Repurposing 18F-FMISO as a PET tracer for translational imaging of nitroreductase-based gene directed enzyme prodrug therapy
Source: Theranostics. 2021 Apr 7;11(12):6044–57. doi: 10.7150/thno.55092 (PMC8058731; doi:10.7150/thno.55092)
Supplement: Supplementary file 1 — Supplementary figures. [file thnov11p6044s1.pdf]

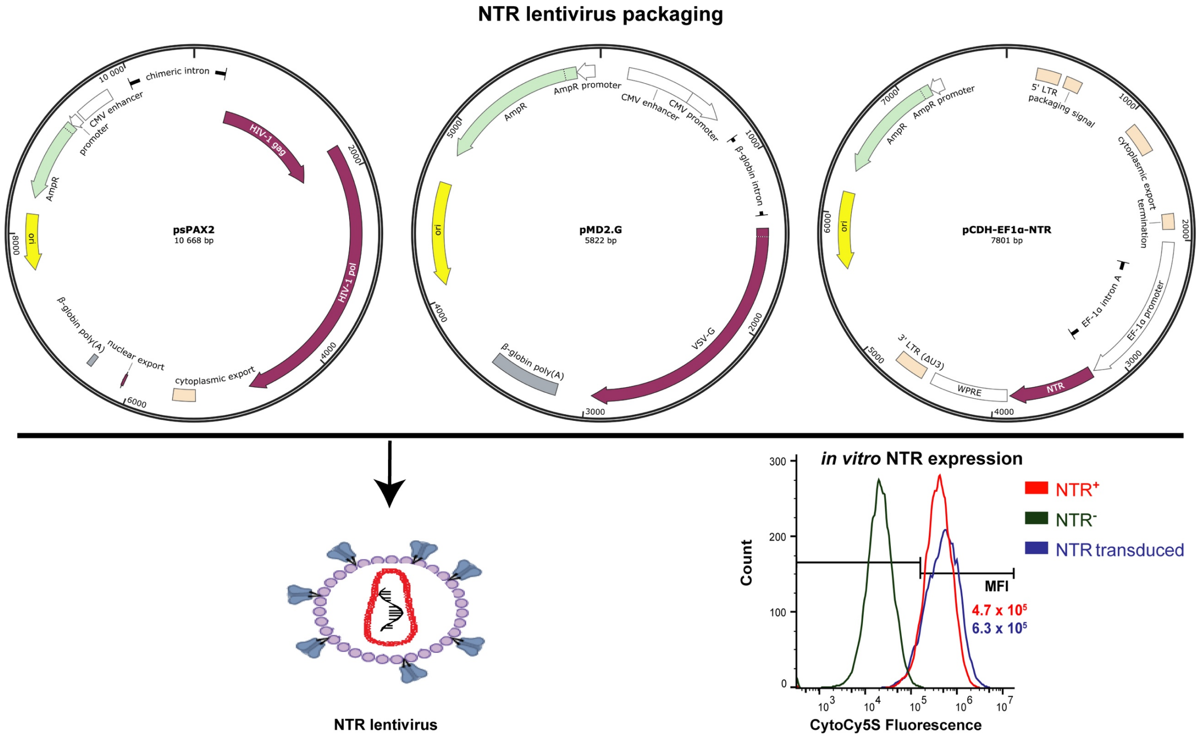

FIGURE S1

NTR lentiviral packaging plasmids. *In vitro* analysis of the levels of expression of NTR in the MDA-MB-231 NTR^{+} cell line and in the MDA-MB-231 after transduction and sorting with the lentiviral NTR particles. Similar levels of expression were observed in both cell lines.

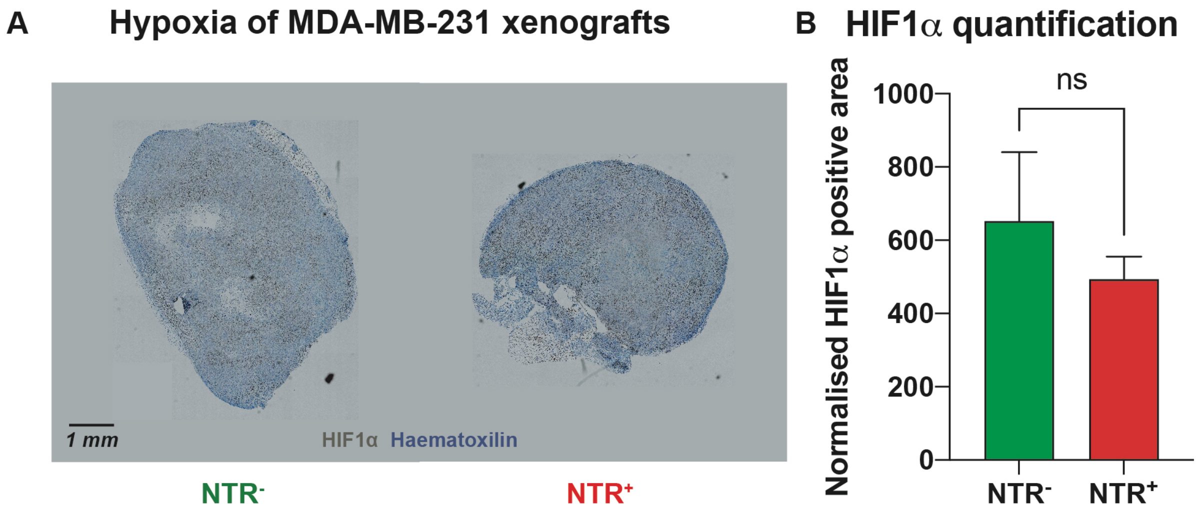

FIGURE S2

(A) Example of HIF1α IHC in NTR$^-$ and NTR$^+$ MDA-MB-231 xenografts. (B) No significant difference was observed in the normalised area positive for HIF1α between NTR$^-$ and NTR$^+$ MDA-MB-231 xenografts (n = 3, per group).

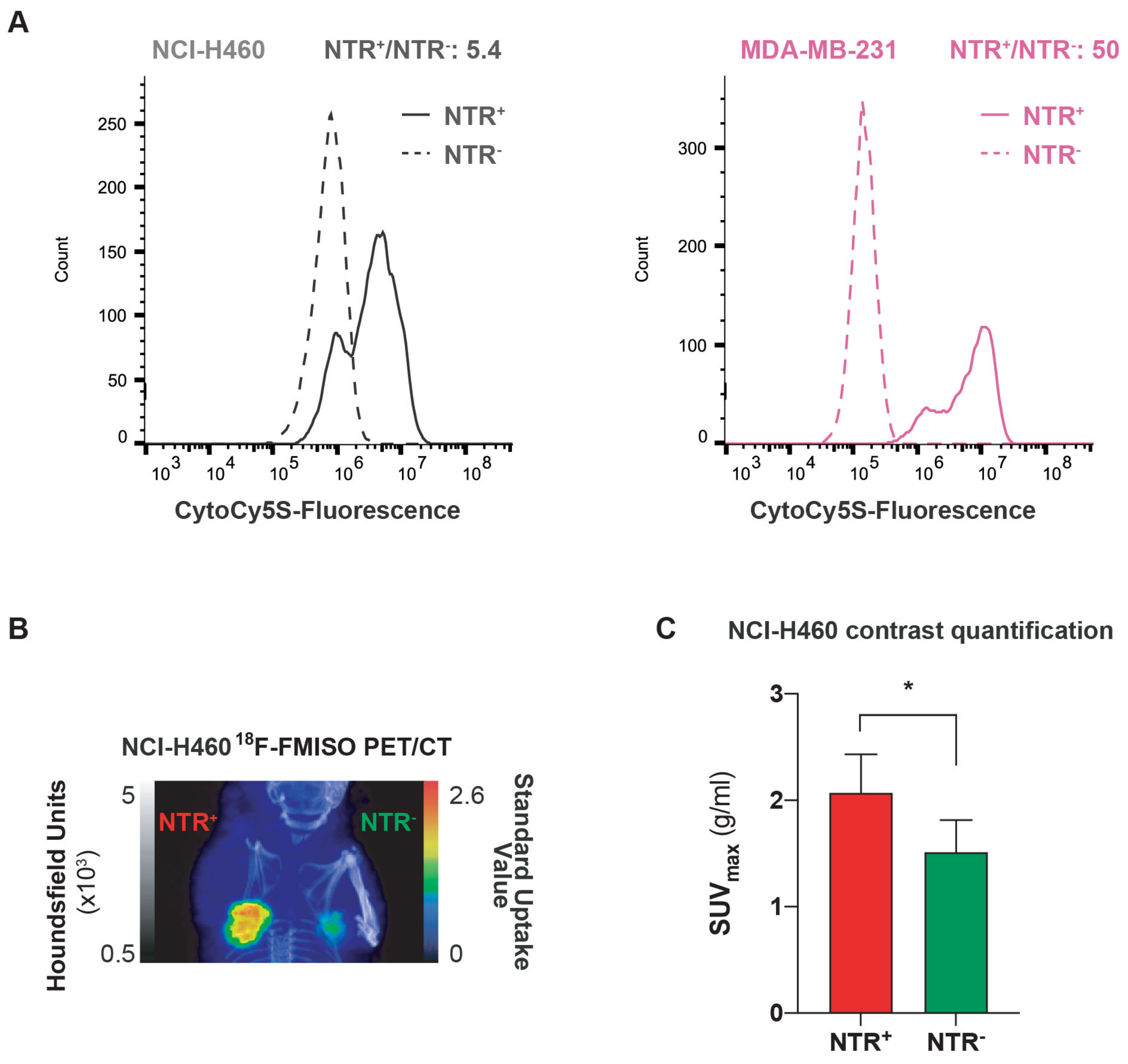

FIGURE S3

(A) NTR^+/NTR^- ratio was notably lower for NCI-H460 than for MDA-MB-231 cell line (B) Representative ^{18}F-FMISO PET/CT MIP images of NCI-H460 xenografts. NTR^- tumour (right flank) and NTR^+ tumour (left flank). (C) SUV_{max} values were significantly higher in NTR^+ xenografts. (*, $p < 0.05$, n = 4)
The p-values are represented as indicated: * $p < 0.05$, ** $p < 0.01$, *** $p < 0.001$ and **** $p < 0.0001$.

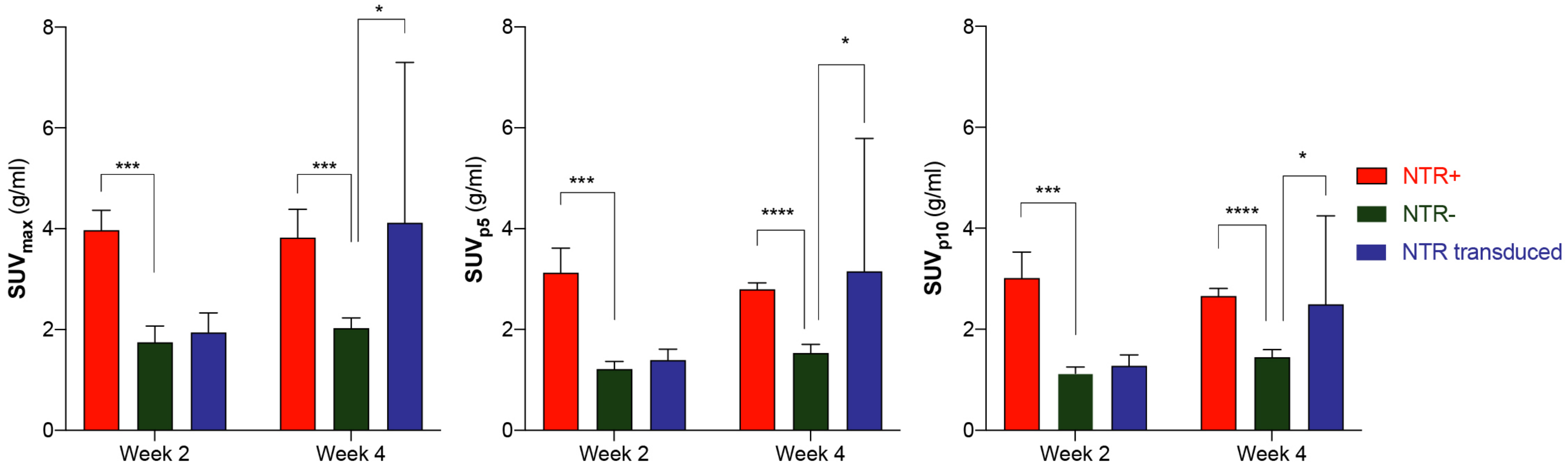

FIGURE S4

SUV_{max}, SUV_{p5} and SUV_{p10} analyses show similar results in the *in vivo* transduced tumours.
The p-values are represented as indicated: * $p < 0.05$, ** $p < 0.01$, *** $p < 0.001$ and **** $p < 0.0001$.

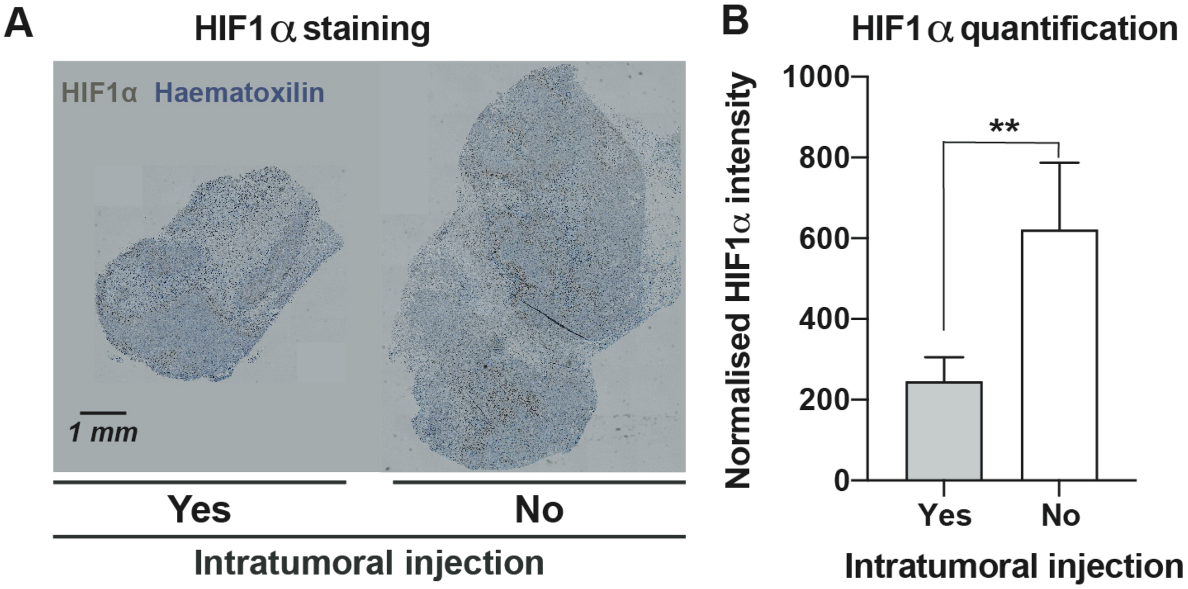

FIGURE S5

(A) The spatial pattern of HIF1α accumulation was similar in both intratumorally injected and non-injected tumours MDA-MB-231 NTR^- xenografts. (B) The normalised HIF1α positive area was significantly lower in intratumorally injected xenografts (n = 4 per group).
